# The association between Vitamin D deficiency and clinical pregnancy rate in IVF patients with different age

**DOI:** 10.3389/fendo.2024.1485238

**Published:** 2025-01-03

**Authors:** Keyan Wang, Fangli Dong, Shuxia Ma, Zhiqin Bu

**Affiliations:** ^1^ Henan Institute of Medical & Pharmaceutical Sciences, Zhengzhou University, Zhengzhou, China; ^2^ Center for Reproductive Medicine, Henan Key Laboratory of Reproduction and Genetics, The First Affiliated Hospital of Zhengzhou University, Zhengzhou, China; ^3^ Reproductive Medical Center, Luoyang Maternal and Child Health Hospital, Luoyang, China

**Keywords:** vitamin D deficiency, endometrial receptivity, homeobox A10, embryo implantation, IVF (*in vitro* fertilization)

## Abstract

**Background:**

The aim of the present study was to investigate the impact of serum VD status on IVF outcomes and to observe the effect of VD deficiency on the expression of the endometrial receptivity marker HOXA10.

**Materials and methods:**

Patients undergoing their first IVF cycles were divided into 3 groups according to VD levels (deficient: <20 ng/mL, insufficient: 20-29.9 ng/mL), and replete ≥30 ng/mL). IVF laboratory parameters, implantation rate, and clinical pregnancy rate were compared among these groups according to patient age (≥ 35 years old and < 35 years old). In addition, the expression of HOXA10 was analyzed using quantitative RT-PCR (qRT-PCR) and western blot in mRNA and protein levels, respectively.

**Results:**

A total of 1459 patients were included. Clinical pregnancy outcomes were significantly worse in vitamin D-deficient patients of advanced age than in other patients. VD status was a predictor of clinical pregnancy according to the multivariate regression model (Deficient: OR = 0.74, 95% CI: 0.59-0.90, P = 0.022; Insufficient: OR = 0.85, 95% CI: 0.70-1.10, P = 0.104; Reference = Replete). However, clinical pregnancy outcomes were comparable among the three groups of young patients. Endometrial tissue was collected from a total of 35 women. HOXA10 expression was significantly lower only in young women in the vitamin D deficiency group. Furthermore, among patients of advanced age, HOXA10 levels were significantly decreased in both vitamin D-deficient and vitamin D-insufficient women.

**Conclusion:**

VD deficiency appears to lead to poorer clinical pregnancy outcomes in patients of advanced age. In the future we can observe whether pregnancy outcomes can be improved in such patients with vitamin D supplementation. In addition, a possible explanation for the worse results may be the detrimental effect of reduced HOXA10 expression on endometrial receptivity.

## Introduction

Vitamin D is a lipid-soluble vitamin that can be obtained from the diet or synthesized in the skin in the presence of sunlight (1). As a type of secosteroid, the main forms of vitamin D include vitamin D2 and vitamin D3. Vitamin D is first absorbed and then hydroxylated to its inactive form, 25-hydroxyvitamin D (calcifediol), in the liver. In the kidney, calfidediol is ultimately hydroxylated by cytochrome P450 family 27 subfamily B member 1 (CYP27B1) to the active form 1,25-dihydroxyvitamin D (calcitriol), which binds to the vitamin D receptor (VDR) and performs many important functions, especially in the female reproductive system (2, 3).

In 2010, a prospective study with a small sample size showed that the serum and follicular fluid levels of vitamin D were strongly correlated. In addition, women with higher vitamin D levels in their serum and follicular fluid are significantly more likely to achieve clinical pregnancy following treatment (4). The association between vitamin D levels and *in vitro* fertilization (IVF) outcomes has been extensively studied. Despite conflicting results, most studies suggest that vitamin D deficiency (VDD) is correlated with adverse IVF pregnancy outcomes (5–7).

The human endometrium is a dynamic tissue that becomes receptive to embryo implantation in a short period (8, 9). Thus, endometrial receptivity is crucial for pregnancy initiation. To date, the molecular regulation of endometrial receptivity has been explored in detail. Among the important regulators of this process, homeobox genes are leading candidates for regulators of endometrial and embryonic development in response to sex steroids (10). In humans, homeobox A10 (HOXA10) expression is markedly increased during the peri-implantation period, and HOXA10 is now considered a molecular marker for endometrial receptivity (11). This gene regulates the expression of other genes involved in cell proliferation, differentiation, and tissue remodeling, making it essential for proper uterine function and implantation. Reduced expression of HOXA10 has been linked to recurrent implantation failure and poor endometrial receptivity. Furthermore, epigenetic changes, such as DNA methylation, can alter HOXA10 expression, influencing reproductive outcomes (12).

Currently, the relationship between VDD and IVF outcome is controversial. Additionally, the mechanism by which VDD affects pregnancy is still unknown. In 2022, a study explored the relationship between endometrial HOXA 10 and Vitamin D levels in PCOS women, and showed that circulating vitamin D levels influence the endometrial HOXA10 gene expression (13). These findings indicate that vitamin D plays a vital role in regulating HOXA10, influencing endometrial receptivity and fertility. Other than that, scarce data exist on the association between VDD and endometrial receptivity. Based on these questions, we aimed to investigate the impact of VDD on IVF outcomes in patients of different ages and to explore the association between VDD and the expression of the endometrial receptivity marker HOXA10 in human uterine tissues.

## Materials and methods

Patients who visited the Reproductive Medicine Center of Luoyang Women’s and Children’s Hospital from June 2015 to December 2022 undergoing first IVF treatment were included. The exclusion criteria were as follows: (1) ovarian stimulation protocols without using GnRH (Gonadotropin-Releasing Hormone) agonist; (2) uterine malformations, intrauterine adhesions and polyps; (3) oocyte or sperm donation cycles; (4) patients received vitamin D supplements before IVF treatment; (5) cycles with important missing data; (6) repeated cycles. As data were deidentified anonymously and all analyses were retrospective, the requirement for informed consent and ethical approvement were waived.

### Criteria for VDD and IVF protocols

Serum vitamin D levels were measured before the patients entered IVF cycles using a chemiluminescence immunoassay (on the first day of Gonadotrophin administration). The intra- and inter-assay coefficients of variation were 8 and 12%, respectively. The patients were divided into 3 groups according to serum vitamin D levels [Institute of Medicine (IOM) Guidelines]: the deficient (<20 ng/mL), insufficient (20-29.9 ng/mL), and replete (≥30 ng/mL) groups.

The ovarian stimulation protocols were GnRH agonist protocols. After pituitary down-regulation, ovarian stimulation was started with a dose of 150-300 IU per day of r-FSH. When at least 3 follicles ≥ 17 mm in diameter or at least 2 follicles ≥18 mm in diameter were detected by ultrasound, 6,500 or 8,500 IU hCG was injected for triggering. Around 37 hours later, oocytes were retrieved. Three or 5 days later, cleavage stage embryos and blastocysts were transferred. Serum hCG levels were measured 14 days after embryo transfer. Clinical pregnancy was defined as observation of gestational sac with heart beat 5 weeks after embryo transfer.

### Human endometrial tissue collection

Endometrial tissue was from IVF patients for endometrial biopsy during luteal phase endometrial injury. Human tissue collection has also been approved by the Ethics Committee of the First Affiliated Hospital of Zhengzhou University on March, 2019 (2019-KY-252). Written informed consent was obtained from all patients. The inclusion criteria mainly included: female age < 42 years, basic FSH < 10 mIU/mL, menstrual cycle 25-35 days, tubal factor or male factor infertility.

Luteal phase was defined as 7 days after ovulation as confirmed by ultrasound examination and serum progesterone elevation. Endometrial injury was performed by the same physician with Pipelle (Disposable Endometrium Suction Tube, S-3.2; Nuode Medical, Jiangxi, China). On day of endometrial injury, serum and endometrial tissue were collected and stored at −20°C and −80°C until analysis after proper preparation.

### RNA isolation and analysis of gene expression by real-time RT-PCR

Total RNA extraction from endometrial tissue was performed by Trizol reagent (Invitrogen Corp, Carlsbad, CA, USA) according to instructions. The RNA was then reverse transcribed in a 20 ml reaction mixture containing 4 μL 5 × Reaction Buffer, 0.5 μL Oligo (dT)18 Primer (100 μM), 0.5 μL And Random Hexamer primer (100 μM), 1 μL Servicebio^®^RT Enzyme Mix, 10 μL Total RNA/mRNA and RNase free water. Quantitative real-time PCR was conducted with a MyiQSingle-Color Real-Time PCR Detection System (Bio-Rad, Hercules, CA, USA). The primer sequences have been described in our previous work (14). The PCR conditions were as follows: initial denaturation at 95°C for 10 min, followed by 40 cycles of 95°C for 15 s, 60°C for 30 s and annealing for 20 s and elongation at 72°C for 20 s. The fold change in the expression of HOXA10 gene was calculated using the 2–ΔΔ CT method. The expression level of target gene was normalized to β-actin mRNA and expressed as n-fold difference relative to the control.

### Western blotting analysis

Tissues were washed with pre-cooled phosphate-buffered saline (PBS) for 2-3 times before being put into lysis buffer containing freshly added protease inhibitors. Solid debris was removed by centrifugation at 12000 rpm for 10 min at 4°C. Proteins and pre-stained molecular weight markers were separated by 10% sodium dodecyl sulfate (SDS) polyacrylamide gel, and were transfer onto polyvinylidene fluoride (PVDF) membranes (0.45 μm, Sigma-Aldrich Corp, Mo, USA). The membranes were blocked in TBST (Tris-buffered saline with 0.5% Triton X-100) containing non-fat milk, and then incubated with primary antibodies anti-HOXA10 (1:1,000; Santa Cruz, CA, USA), anti-actin (1:2,000; Santa Cruz, CA, USA) overnight at 4°C. Protein bands were visualized using an enhanced chemiluminescence detection system (Bio-Rad) and measured using a quantitative scanning densitometer. The relative level of HOXA10 was analyzed and compared using ImageJ software.

### Statistical analysis

The normality of all measured variables was firstly evaluated (Skewness and Kurtosis test). The data were expressed as mean ± standard deviation (SD), or median (25th, 75th percentile), and were analyzed using Package for the Social Sciences 21.0 (SPSS, Chicago, IL, USA). For normal distribution data, differences between groups were compared by Student’s t-test. If data were not with normal distribution, either Mann-Whitney test was used or data were log transformed prior to further statistical analysis. Patient basic parameters and clinical pregnancy outcomes were analyzed in the three VD groups according to different ages. Univariate and multivariate logistic regression analysis were also used to explore the impact of VDD on clinical pregnant rates. P < 0.05 was considered to be statistically significant.

## Results

A total of 1459 patients who underwent their first IVF cycles and were treated with the GnRH agonist protocol were included. There were 429, 726, and 274 patients in the vitamin D-deficient, vitamin D-insufficient, and vitamin D-replete groups, respectively. The basic characteristics of the patients in these three groups are presented in **Table 1**. Although VD levels differed among the three groups, age, BMI, basic FSH levels, and infertility diagnosis were comparable between patients with and without VDD.

**Table 1:** Basic parameters in patients with different Vitamin D status.

Characteristics	Deficient (<20 ng/mL)	Insufficient (20-29.9 ng/mL)	Replete (≥ 30 ng/mL)	*P* value
No. of cycles	459	726	274	
Age (years)	33.1± 5.2	32.8 ± 5.8	32.2± 5.1	0.479
BMI (kg/m^2^)	23.9± 3.4	23.8± 3.2	23.2± 3.8	0.820
Basal FSH (mIU/mL)	7.4± 3.5	7.3± 2.2	7.2± 3.3	0.140
AFC (n)	11 (8, 18)	12 (9, 18)	13 (8, 18)	0.258
25, (OH)D_3_ (ng/mL)	17.2± 1.8	25.0± 2.8	32.3± 2.7	< 0.001
Infertility Diagnosis				
Tubal factor	155 (45.19)	227 (46.51)	80 (43.24)	
Ovulation disorder	62 (18.09)	96 (19.67)	32 (17.30)	0.350
Male factor	82 (23.91)	120 (24.59)	47 (25.41)	
Others	44 (12.83)	45 (9.23)	26 (14.05)	
Seasons				0.625
Spring	110 (23.97)	160 (22.04)	62 (22.63)	
Summer	149 (32.46)	221 (30.44)	93 (33.94)	
Autumn	113 (24.62)	190 (26.17)	61 (22.26)	
Winter	87 (18.95)	155 (21.35)	58 (21.17)	

BMI, body mass index; FSH, follicle stimulation hormone; AMH, anti-Müllerian hormone; AFC, antral follicle count. Data presented as means ± SD, median (25th, 75th percentile), n or n (%).

**Figure 1:**
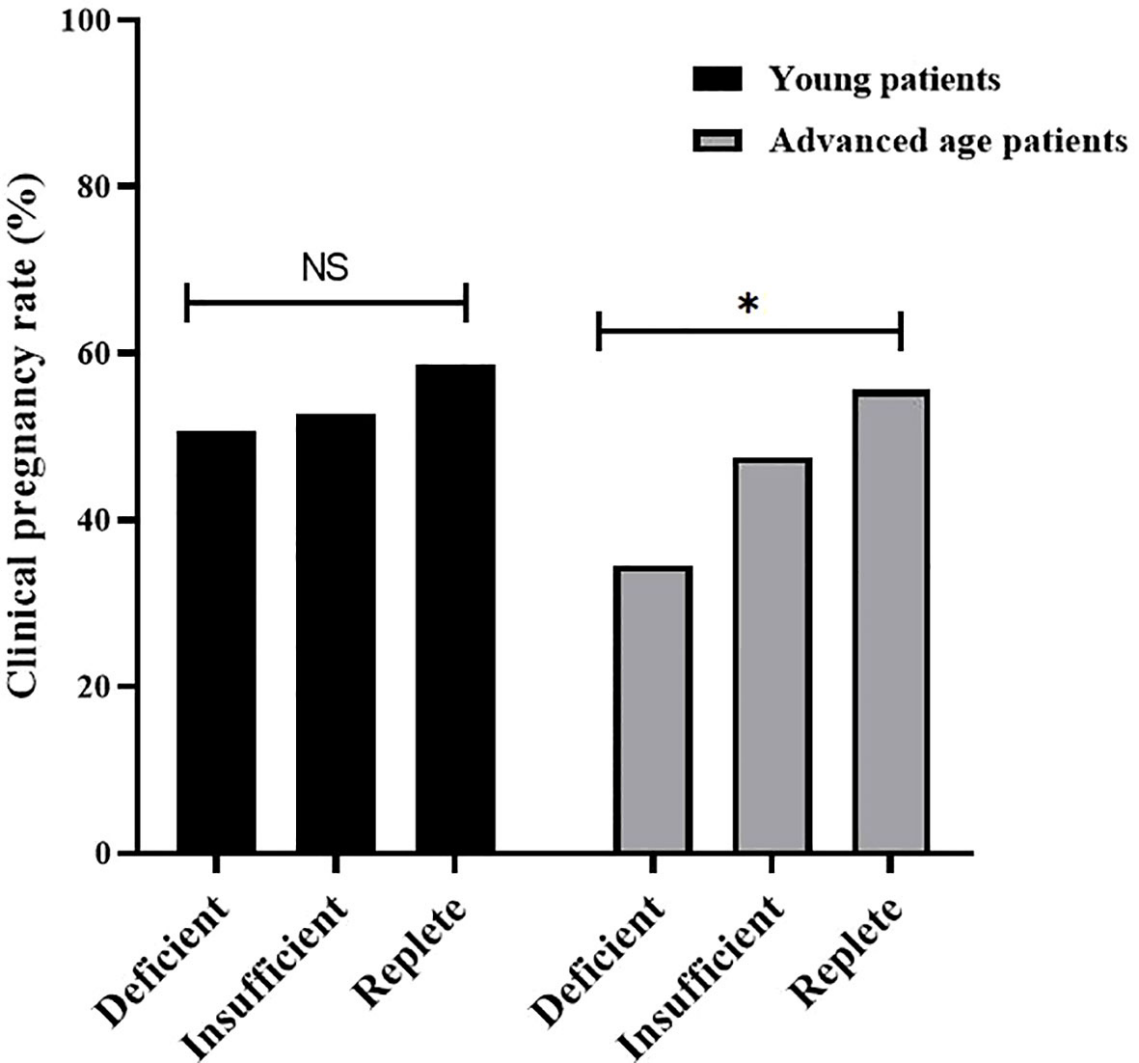
Clinical pregnancy rate of patients with different vitamin D status (Replete, Insufficient, Deficient) in young patients and advanced age patients. Clinical pregnancy rates were significantly different among vitamin D-deficient, vitamin D-insufficient, and vitamin D-replete group in advanced age patients, but not in young patients. NS, not significant; * P < 0.05.

As shown in **Table 2**, to compare the IVF laboratory results and clinical pregnancy outcomes among the three groups, all patients were first divided into young (< 35 years old) and advanced age (≥ 35 years old) groups. Regardless of patient age, laboratory outcomes, mainly including the number of oocytes retrieved, number of 2PNs (Pronuclei), number of high-quality embryos, and number of transferrable embryos, were similar among the three groups. In addition, for clinical pregnancy outcomes, implantation rates and clinical pregnancy rates tended to be greater in VD-replete women than in VD-deficient and VD-insufficient women of a young age, yet the differences were not statistically significant. However, in patients of advanced age, clinical pregnancy outcomes were significantly different among vitamin D-deficient, vitamin D-insufficient, and vitamin D-replete patients (**Figure 1**).

**Table 2:** Comparison of laboratory parameters in patients with different Vitamin D status according to age.

	Young patients				Advanced age patients			
	< 35 years old				≥ 35 years old			
	Deficient (<20 ng/mL)	Insufficient (20-29.9 ng/mL)	Replete (≥30 ng/mL)	*P*	Deficient (<20 ng/mL)	Insufficient (20-29.9 ng/mL)	Replete (≥30 ng/mL)	*P*
Number of cycles	312	482	176		147	244	98	
Duration of Gn used (d)	11.0± 2.0	11.1± 2.1	10.9± 2.0	0.142	12.8± 2.3	13.1± 2.5	12.4± 2.2	0.158
Peak E_2_ level (pg/ml)	3686.0 ± 2524.8	3667.6 ± 1975.4	3684.7± 1787.0	0.682	1615.7 ± 1725.6	1588.6 ± 1586.2	1769.0 ± 1755.2	0.370
Endometrial thickness on trigger day (mm)	11.5± 2.4	11.7± 2.6	11.7± 2.5	0.811	10.5 ± 2.0	10.6 ± 2.4	10.6 ± 2.2	0.921
No. of oocytes retrieved (n)	12 (7, 19)	13 (7, 19)	12 (7, 18)	0.357	8 (3, 12)	9 (7, 14)	9 (7, 15)	0.347
No. of 2PN (n)	9 (5, 11)	8 (5, 11)	8 (5, 11)	0.813	6 (3, 9)	6 (3, 10)	7 (4, 10)	0.733
2PN fertilization rate (%)	83.3(69.2, 100.0)	83.3 (71.4, 100.0)	83.3(67.0, 100.0)	0.999	81.3(66.3, 100.0)	80.8 (71.8, 100.0)	79.3(64.1, 100.0)	0.999
No. of transferable embryos (n)	7 (5, 10)	7(4, 11)	8 (5, 11)	0.949	4 (2, 6)	4 (2, 5)	5 (2, 5)	0.910
No. of good-quality embryos (n)	3 (1, 6)	3(1, 6)	3 (1, 6)	0.988	1 (1,2)	1(1, 2)	1 (1, 2)	0.981
No. of transfer cycles	150	254	87		81	124	61	
No. of embryos transferred	1 (1, 2)	1 (1, 2)	1 (1, 2)	0.999	1 (1, 2)	1 (1, 2)	1 (1, 2)	0.999
Implantation Rate % (n/N)	36.7 (80/218)	38.2 (139/363)	45.2 (57/126)	0.270	27.0 (33/122)	34.4 (64/186)	44.5 (41/92)	0.028
Early miscarriage rate % (n/N)	10.67 (16/150)	9.06 (23/254)	9.20 (8/87)	0.851	19.75 (16/81)	14.52 (18/124)	16.39 (10/61)	0.201
Ectopic pregnancy rate % (n/N)	0.67 (1/150)	1.18 (3/254)	1.15 (1/87)	0.943	0.00 (0/81)	1.61 (2/124)	3.28 (2/61)	0.866
Clinical pregnancy rate, % (n/N)	50.7 (76/150)	52.7 (134/254)	58.6 (51/87)	0.488	34.6 (28/81)	47.6 (59/124)	55.7 (34/61)	0.035

Gn, gonadotropin; d days; E_2,_estradiol; LH, luteinizing hormone; IVF, *in vitro* fertilization; ICSI, intracytoplasmic sperm injection; 2PN, 2 pronuclei. Data presented as means ± SD, median (25th, 75th percentile), n or n (%).

**Table 3** shows the results of logistic regression analysis of risk factors for clinical pregnancy with both univariate and multivariate models. According to the univariate regression analysis, female age, endometrial thickness on the trigger day, number of high-quality embryos, and number of embryos transferred were associated with clinical pregnancy status regardless of patient age. However, in young patients, after adjusting for cofounding factors, only female age, endometrial thickness on the trigger day, number of high-quality embryos, and number of embryos transferred were risk factors for clinical pregnancy. Serum VD status was not a predictor of pregnancy outcomes. In women of advanced age, VD status was significantly related to clinical pregnancy status according to univariate logistic regression analysis. In addition, the relationship between VD status and pregnancy outcomes did not change after adjusting for cofounding factors in the multivariate regression model.

**Table 3:** Logistic regression analysis of risk factors for clinical pregnancy in patients undergoing fresh embryo transfer.

Parameters	Univariable regression		Multivariable regression		Univariable regression		Multivariable regression	
	OR (95% CI)	*P*	aOR^a^ (95% CI)	*P*	OR (95% CI)	*P*	aOR^b^ (95% CI)	*P*
Female age	0.87 (0.66-0.90)	0.012	0.75 (0.61-0.88)	0.001	0.74 (0.60-0.89)	0.001	0.71 (0.61-0.84)	0.001
BMI (Kg/m^2^)	0.97 (0.88-1.07)	0.450	–	–	1.04 (0.84-1.10)	0.154	–	–
Duration of Gn used	1.08 (0.87-1.14)	0.547	–	–	0.97 (0.80-1.11)	0.620	–	–
Peak E_2_ level (pg/ml)	1.00 (0.99-1.01)	0.917	–	–	1.00 (0.99-1.01)	0.856	–	–
Endometrial thickness on trigger day (mm)	1.14 (1.07-1.25)	0.031	1.15 (1.10-1.26)	0.014	1.18 (1.08-1.28)	0.014	1.20 (1.11-1.40)	0.028
No. of oocytes retrieved	1.26 (0.91-1.42)	0.067	–	–	1.24 (1.14-1.18)	0.048	1.14 (0.91-1.24)	0.063
No. of 2PN	1.28 (0.94-1.37)	0.077	–	–	1.15 (0.85-1.35)	0.089	–	–
No. of transferable embryos	1.20 (1.14-1.55)	0.020	1.12 (0.98-1.15)	0.090	1.13 (0.92-1.24)	0.120	–	–
No. of good-quality embryos	1.33 (1.07-1.46)	0.001	1.30 (1.10-1.44)	0.004	1.35 (1.17-1.50)	0.028	1.36 (1.20-1.52)	0.017
No. of embryos transferred	1.40 (1.21-1.63)	0.001	1.42 (1.22-1.58)	0.001	1.55 (1.20-1.67)	0.001	1.58 (1.22-1.70)	0.001
Vitamin D status		0.488				0.035		
Deficient	0.81 (0.70-1.06)	0.063	–	–	0.78 (0.60-0.94)	0.037	0.74 (0.59-0.90)	0.022
Insufficient	0.86 (0.74-1.02)	0.078	–	–	0.83 (0.65-1.05)	0.088	0.85 (0.70-1.10)	0.104
Replete	Reference				Reference			

BMI, body mass index; OR, adds ratio; CI, confidential interval; Gn, gonadotrophin; PN, Pronuclei.

a Adjusted for female age, endometrial thickness on trigger day, No. of transferable embryos, No. of good-quality embryos, No. of embryos transferred.

b Adjusted for female age, endometrial thickness on trigger day, No. of oocyte retrieved, No. of good-quality embryos, No. of embryos transferred, Vitamin D status.

Expression of HOXA10 in endometrial tissue from women undergoing IVF with or without vitamin D deficiency.

**Figure 2:**
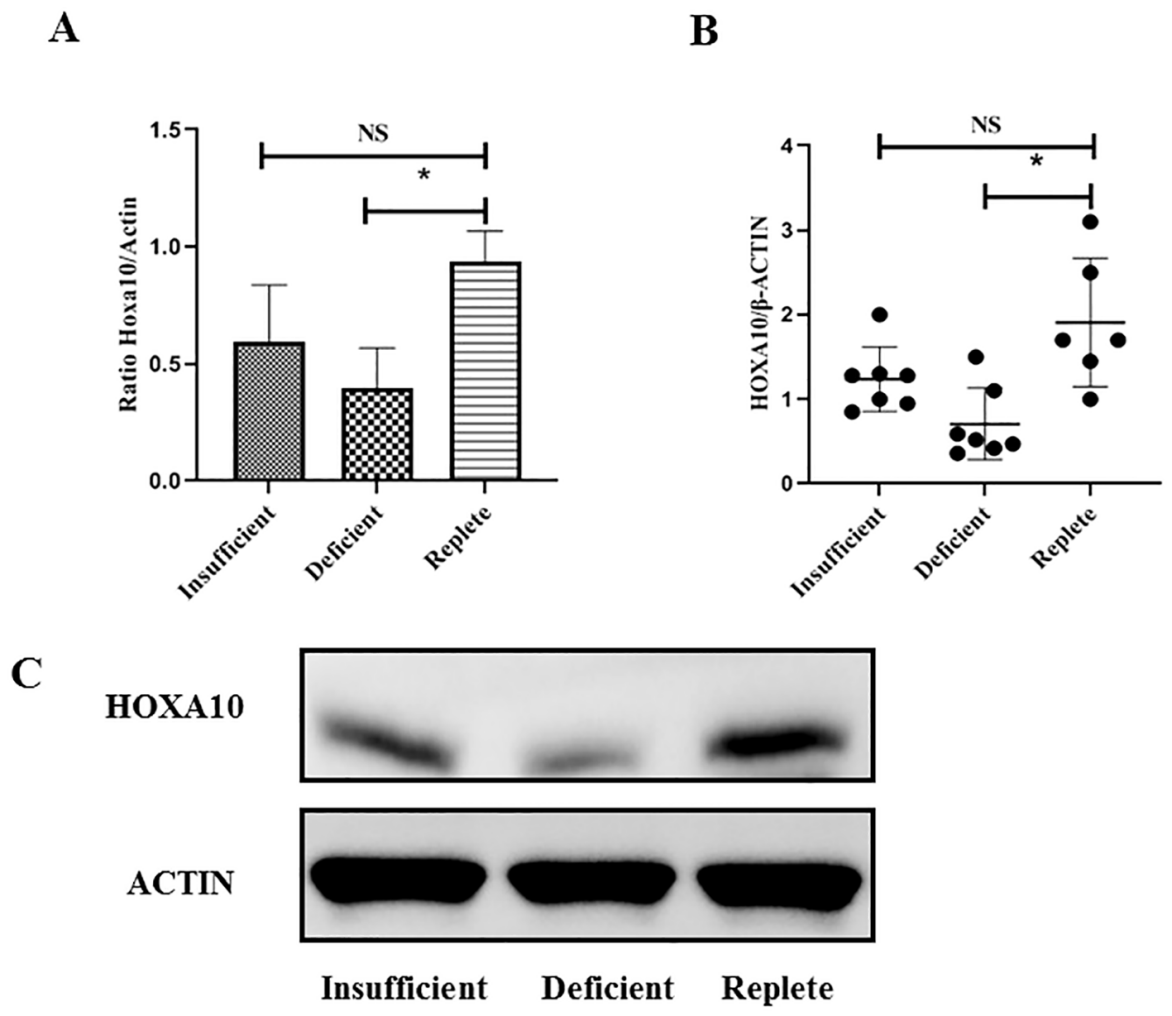
Expression of HOXA10 in endometrial tissue from young age women (< 35 years old) during implantation window according to vitamin D status (Replete, Insufficient, Deficient). The expression of HOXA10 is significantly decreased at mRNA **(A)** and protein **(B, C)** levels in uteri tissue from women with vitamin D Deficient (*N* = 7) when compared with those with vitamin D Replete (*N* = 6). The expression between vitamin D Insufficient Group (*N* = 7) and vitamin D Replete Group is comparable. NS, not significant; HOXA10, homeobox A10; * *P* < 0.05.

**Figure 3:**
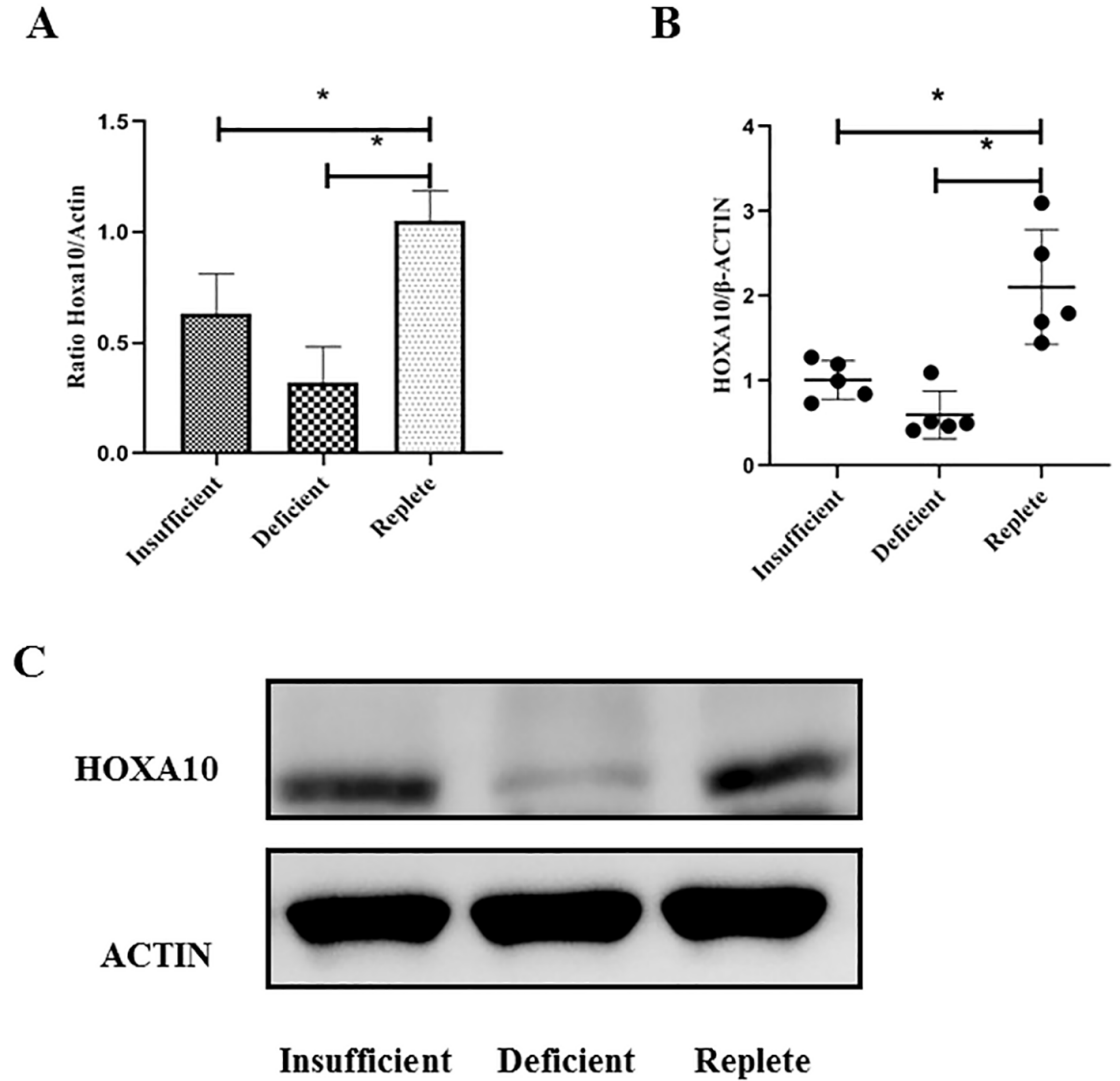
Expression of HOXA10 in endometrial tissue from advanced age women (≥ 35 years old) during implantation window according to vitamin D status (Replete, Insufficient, Deficient). The expressions of HOXA10 are significantly decreased at mRNA **(A)** and protein **(B, C)** levels in uteri tissue from women both with vitamin D Deficient (*N* = 5), with vitamin D Insufficient (*N* = 5), when compared with those with vitamin D Replete (*N* = 5). HOXA10, homeobox A10; * *P* < 0.05.

Endometrial tissue was obtained from women undergoing luteal phase endometrial injury during IVF treatment. Endometrial tissue was collected from a total of 20 young patients (6, 7, and 7 patients in the vitamin D-replete, vitamin D-insufficient, and vitamin D-deficient groups, respectively) and 15 advanced age patients (5, 5, and 5 patients in the vitamin D-replete, vitamin D-insufficient, and vitamin D-deficient groups, respectively). As shown in **Figure 2**, the expression of HOXA10 at the mRNA and protein levels in endometrial tissue was measured using RT-PCR in young women. Compared with those in women who were vitamin D-sufficient, HOXA10 mRNA expression and protein levels were significantly lower only in the endometria of women in the vitamin D-deficient group (P< 0.05) but not in those of women in the vitamin D-insufficient group. However, in patients of advanced age, as shown in **Figure 3**, HOXA10 levels were significantly decreased in the endometrial tissue of vitamin D-deficient patients (2.15 ± 0.39 vs 0.58 ± 0.11; P = 0.029) and the endometrial tissue of vitamin D-insufficient patients (2.15 ± 0.39 vs 1.05 ± 0.24; P < 0.034).

## Discussion

VDD is recognized to be prevalent worldwide. VDD has been associated with increased risk of hypertension, infectious diseases, autoimmune diseases, and reproductive system disorders, including preeclampsia, miscarriage, and infertility (15). First, the current study revealed a positive association between VDD and embryo implantation outcome in IVF patients. However, VD status seemed to have no impact on embryo quality. In addition, decreased expression of the endometrial receptivity marker HOXA10 was observed in female VDD patients compared with vitamin D-replete women of advanced age but not in young women.

Recently, two studies in mice by Zarnani et al. also showed that vitamin D receptor mRNA was expressed in the endometrium, ovary, and fallopian tubes throughout the estrous cycle and increased in expression in the estrus phase compared with the other phases (16). In addition, during pregnancy, vitamin D receptor mRNA and protein are also expressed in the decidua, placenta and ovary (17), indicating that vitamin D plays important roles in female reproductive function.

However, in contrast to data from animals, results regarding whether vitamin D status impacts IVF outcomes in humans are limited (18). Implantation is a complex procedure involving several factors, and has a critical effect on this process. It is reasonable to explore the impact of VD on pregnancy outcomes in IVF patients of different ages. As shown in this study, IVF laboratory characteristics, especially the number of high-quality embryos, were comparable between patients with different VD statuses. The embryo implantation rate and clinical pregnancy rate were significantly lower in the VD-deficient group than in the VD-replete group among women aged ≥ 35 years but not among women aged < 35 years.

Thus, what is the specific mechanism by which VDD adversely affects IVF outcomes? Rudick and colleagues demonstrated that VDD is associated with lower pregnancy rates in non-Hispanic white women, but ovarian stimulation parameters or embryo quality markers are the same among women with VDD, VD insufficiency, and sufficient VD, which indicates that the adverse impact of VDD on pregnancy may be mediated through the endometrium (19). To support this conclusion, the same study group further explored pregnancy rates among oocyte donor recipients with different vitamin D statuses and reported that a vitamin D concentration <30 ng/mL was associated with lower pregnancy rates (20). More importantly, a recent clinical study in 1883 IVF women showed that serum vitamin D levels were not significantly related to embryo quality in the cleavage or blastocyst stage (21). Since oocyte and embryo quality were comparable among the groups and not affected by vitamin D levels, it is speculated that endometrial receptivity is compromised, as low pregnancy rates are observed in women with VDD. However, a recent study in 2021 indicated that neither total nor free 25(OH)D serum concentration was associated with pregnancy outcomes, such as implantation rate, clinical pregnancy rate, and ongoing pregnancy rate, etc. (22). The reason our results differ from those findings may be due to differences in the study populations and the timing of vitamin D measurement. We only observed positive results in advanced age women, but not in young patients, either.

Indeed, vitamin D receptors are expressed in the endometrium, and vitamin D is considered a natural regulator of the reproductive and immune system (23). In addition, the expression of vitamin D receptors is significantly different in the endometrial secretory phase compared to the proliferative phase, suggesting that the vitamin D-VDR system plays a role in the development of endometrial receptivity (24). In our study, a strong positive association was found between vitamin D levels and the expression of the endometrial receptivity marker HOXA10 in human uterine tissue. This phenomenon was consistent with the findings of another study involving human myelomonocytic cells and human endometrial stromal cells. Their data also showed direct regulation of HOXA10 expression by vitamin D (25). Thus, it is reasonable to speculate that VDD adversely affects implantation by reducing endometrial receptivity.

However, the specific mechanism by which vitamin D deficiency affects HOXA10 expression and subsequently impacts embryo implantation remains unclear (26). Current studies on this topic are predominantly observational, and the precise pathways involved have not yet been elucidated. This warrants further studies drawing on knowledge from fields like immunology or endocrinology could offer more insight into the biological pathways involved. In the future, studies focusing on cytokine profiles (IL-6, TNF-α, etc.) and their relation to endometrial receptivity can be conducted.

One of the strengths of the current study is that IVF laboratory and clinical parameters and the expression of the endometrial receptivity marker HOXA10 were explored. These data not only reveals whether VD status affects IVF clinical outcomes but also provides some insights into the possible underlying mechanism. In addition, we divided patients into young and advanced age groups for the first time before analysis. Interestingly, VD status significantly impacted embryo implantation only in women with a worse prognosis. Overall, the expression of endometrial receptivity markers seems to be decreased in vitamin D-deficient female patients regardless of age. The reason why only women aged ≥ 35 years experienced dramatically poorer pregnancy outcomes is that embryos from these patients are more vulnerable to other factors, such as vitamin D status. However, several limitations also exist. Due to the retrospective nature of this study, some social factors related to vitamin D concentration, such as lifestyle, education, income, diet, and nutritional status, cannot be fully obtained. As presented in a study by Hocher et al. in 2014, all these factors mentioned above have influence on vitamin D status (27). Therefore, these biases may still exist. Second, only IVF outcomes after fresh embryo transfer cycles were evaluated in this study, and embryo quality could also reflect the outcomes of subsequent frozen-thawed transfers from the same oocyte retrieval cycles. In addition, it would be better to assess the effect of vitamin D deficiency on IVF outcomes by using data from intervention studies focusing on vitamin D supplementation in the future. Moreover, as the study only included 15 advanced age patients, the sample size is relatively small, resulting in limited statistical power. Therefore, caution should be exercised when interpreting the conclusions to avoid biases or uncertainties caused by insufficient sample size. We hope to include more patients in future studies and conduct multicenter prospective clinical research, also including frozen thawed embryo transfer cycles, to further explore the impact of VDD on pregnancy outcomes. Last but not least, we should also be caution that, like many other similar studies, total vitamin D and free vitamin D cannot be separated in this study. Unlike total vitamin D, the free one is the small, unbound, biologically active portion, which is crucial for reproductive processes, including endometrial receptivity, and embryo implantation (28, 29).

## Conclusion

In summary, our study showed that during IVF treatment, vitamin D deficiency appears to result in significantly worse clinical pregnancy outcomes in patients of advanced age but not in young patients. A possible explanation for the worse results may be the detrimental effect of reduced HOXA10 expression on endometrial receptivity. These findings suggest that vitamin D supplementation for advanced age patients may improve clinical pregnancy outcomes in IVF treatments. Further research is needed to confirm this potential benefit and to establish optimal supplementation strategies for this patient population.

## Data Availability

The original contributions presented in the study are included in the article/supplementary material. Further inquiries can be directed to the corresponding authors.

## References

[1] Gould JF Gibson RA Green TJ Makrides M. A systematic review of vitamin D during pregnancy and postnatally and symptoms of depression in the antenatal and postpartum period from randomized controlled trials and observational studies. NUTRIENTS. (2022) 14:1–17. doi: 10.3390/nu14112300 PMC9183028 35684101

[2] Nikolac GN Unic A Miler M Pavicic T Culej J Bolanca I. In sickness and in health: pivotal role of vitamin D. Biochem Med (Zagreb). (2020) 30:20501. doi: 10.11613/BM.2020.020501 PMC7271749 32550812

[3] Phillips EA Hendricks N Bucher M Maloyan A. Vitamin D supplementation improves mitochondrial function and reduces inflammation in placentae of obese women. Front Endocrinol (Lausanne). (2022) 13:893848. doi: 10.3389/fendo.2022.893848 35712242 PMC9195071

[4] Ozkan S Jindal S Greenseid K Shu J Zeitlian G Hickmon C. Replete vitamin D stores predict reproductive success following *in vitro* fertilization. FERTIL STERIL. (2010) 94:1314–9. doi: 10.1016/j.fertnstert.2009.05.019 PMC2888852 19589516

[5] Arnanz A Garcia-Velasco JA Neyro JL. Calcifediol (25OHD) deficiency and its treatment in women’s health and fertility. NUTRIENTS. (2022) 14:1–18. doi: 10.3390/nu14091820 PMC9103696 35565788

[6] Chu J Gallos I Tobias A Tan B Eapen A Coomarasamy A. Vitamin D and assisted reproductive treatment outcome: a systematic review and meta-analysis. Hum Reprod. (2018) 33:65–80. doi: 10.1093/humrep/dex326 29149263

[7] Tunccan E Mohri P Dikec M Karaawi F Kazaz E Kocatepe C. Effects of preconceptional vitamin D levels on *in vitro* fertilization outcomes in infertile patients with polycystic ovary syndrome: A retrospective cohort study. J Obstet Gynaecol Res. (2024) 50:2121–30. doi: 10.1111/jog.v50.11 39329337

[8] Evans J Salamonsen LA Winship A Menkhorst E Nie G Gargett CE. Fertile ground: human endometrial programming and lessons in health and disease. Nat Rev Endocrinol. (2016) 12:654–67. doi: 10.1038/nrendo.2016.116 27448058

[9] Shekibi M Heng S Nie G. MicroRNAs in the regulation of endometrial receptivity for embryo implantation. Int J Mol Sci. (2022) 23:1–16. doi: 10.3390/ijms23116210 PMC9181585 35682889

[10] Ekanayake DL Malopolska MM Schwarz T Tuz R Bartlewski PM. The roles and expression of HOXA/Hoxa10 gene: A prospective marker of mammalian female fertility? Reprod Biol. (2022) 22:100647. doi: 10.1016/j.repbio.2022.100647 35550944

[11] Das SK. Regional development of uterine decidualization: molecular signaling by Hoxa-10. Mol Reprod Dev. (2010) 77:387–96. doi: 10.1002/mrd.21133 PMC4267754 19921737

[12] Wei W Wang N Zhu Y Liao M Wang B Du T. GM-CSF improves endometrial receptivity in a thin endometrium rat model by upregulating HOXA10. Mol Hum Reprod. (2023) 30:1–9. doi: 10.1093/molehr/gaad042 38011650

[13] Shilpasree AS Kulkarni VB Shetty P Bargale A Goni M Oli A. Induction of endometrial HOXA 10 gene expression by vitamin D and its possible influence on reproductive outcome of PCOS patients undergoing ovulation induction procedure. Indian J Endocrinol Metab. (2022) 26:252–8. doi: 10.4103/ijem.ijem_90_22 PMC9555374 36248036

[14] Xu B Geerts D Bu Z Ai J Jin L Li Y. Regulation of endometrial receptivity by the highly expressed HOXA9, HOXA11 and HOXD10 HOX-class homeobox genes. Hum Reprod. (2014) 29:781–90. doi: 10.1093/humrep/deu004 24549215

[15] Hart RJ. Nutritional supplements and IVF: an evidence-based approach. Reprod BioMed Online. (2024) 48:103770. doi: 10.1016/j.rbmo.2023.103770 38184959

[16] Zarnani AH Shahbazi M Salek-Moghaddam A Zareie M Tavakoli M Ghasemi J. Vitamin D3 receptor is expressed in the endometrium of cycling mice throughout the estrous cycle. FERTIL STERIL. (2010) 93:2738–43. doi: 10.1016/j.fertnstert.2009.09.045 19896660

[17] Shahbazi M Jeddi-Tehrani M Zareie M Salek-Moghaddam A Akhondi MM Bahmanpoor M. Expression profiling of vitamin D receptor in placenta, decidua and ovary of pregnant mice. PLACENTA. (2011) 32:657–64. doi: 10.1016/j.placenta.2011.06.013 21764449

[18] Cozzolino M Busnelli A Pellegrini L Riviello E Vitagliano A. How vitamin D level influences *in vitro* fertilization outcomes: results of a systematic review and meta-analysis. FERTIL STERIL. (2020) 114:1014–25. doi: 10.1016/j.fertnstert.2020.05.040 33012554

[19] Rudick B Ingles S Chung K Stanczyk F Paulson R Bendikson K. Characterizing the influence of vitamin D levels on IVF outcomes. Hum Reprod. (2012) 27:3321–7. doi: 10.1093/humrep/des280 22914766

[20] Rudick BJ Ingles SA Chung K Stanczyk FZ Paulson RJ Bendikson KA. Influence of vitamin D levels on *in vitro* fertilization outcomes in donor-recipient cycles. FERTIL STERIL. (2014) 101:447–52. doi: 10.1016/j.fertnstert.2013.10.008 24210230

[21] Jiang L Ji L Song J Qian K. The effect of serum vitamin D levels in couples on embryo development and clinical outcomes. Reprod BioMed Online. (2019) 38:699–710. doi: 10.1016/j.rbmo.2018.12.036 30772193

[22] Cai S Li J Zeng S Hu L Peng Y Tang S. Impact of vitamin D on human embryo implantation-a prospective cohort study in women undergoing fresh embryo transfer. FERTIL STERIL. (2021) 115:655–64. doi: 10.1016/j.fertnstert.2020.09.005 33039126

[23] Eller A Ejzenberg D Monteleone P Soares JJ Baracat EC. Vitamin D and *in vitro* fertilization: a systematic review. J Assist Reprod Genet. (2023) 40:735–43. doi: 10.1007/s10815-023-02767-2 PMC10224880 36884205

[24] Guo J Liu S Wang P Ren H Li Y. Characterization of VDR and CYP27B1 expression in the endometrium during the menstrual cycle before embryo transfer: implications for endometrial receptivity. Reprod Biol Endocrinol. (2020) 18:24. doi: 10.1186/s12958-020-00579-y 32183826 PMC7079352

[25] Du H Daftary GS Lalwani SI Taylor HS. Direct regulation of HOXA10 by 1,25-(OH)2D3 in human myelomonocytic cells and human endometrial stromal cells. Mol Endocrinol. (2005) 19:2222–33. doi: 10.1210/me.2004-0336 15905361

[26] Zhou X Wu X Luo X Shao J Guo D Deng B. Effect of vitamin D supplementation on *in vitro* fertilization outcomes: A trial sequential meta-analysis of 5 randomized controlled trials. Front Endocrinol (Lausanne). (2022) 13:852428. doi: 10.3389/fendo.2022.852428 35370977 PMC8969598

[27] Reichetzeder C Chen H Foller M Slowinski T Li J Chen YP. Maternal vitamin D deficiency and fetal programming–lessons learned from humans and mice. Kidney Blood Press Res. (2014) 39:315–29. doi: 10.1159/000355809 25300533

[28] Tsuprykov O Chen X Hocher CF Skoblo R Lianghong Y Hocher B. Why should we measure free 25(OH) vitamin D? J Steroid Biochem Mol Biol. (2018) 180:87–104. doi: 10.1016/j.jsbmb.2017.11.014 29217467

[29] Zeng S Chu C Doebis C von Baehr V Hocher B. Reference values for free 25-hydroxy-vitamin D based on established total 25-hydroxy-vitamin D reference values. J Steroid Biochem Mol Biol. (2021) 210:105877. doi: 10.1016/j.jsbmb.2021.105877 33741448

